# Research on three-dimensional path planning of unmanned aerial vehicle based on improved Whale Optimization Algorithm

**DOI:** 10.1371/journal.pone.0316836

**Published:** 2025-02-24

**Authors:** HaoCheng Wang, ZeXian Hao, Yu Zhang

**Affiliations:** Northeast Forestry University, Harbin, China; Hanshan Normal University, CHINA

## Abstract

Addressing the insufficient optimization performance in drone 3D path planning and the issues of inadequate optimization precision and tendency to fall into local optima in the existing Whale Optimization Algorithm (WOA), this paper proposes a drone 3D path planning method based on an improved Whale Optimization Algorithm (CSRD-WOA). Firstly, to enhance the search efficiency and fitness accuracy of the Whale Algorithm, the Cuckoo Search and Random Differential Strategy were introduced and compared with the traditional Particle Swarm Optimization algorithm, Whale Algorithm, and Cuckoo Search Algorithm. Experimental results demonstrate that the CSRD-WOA algorithm improves global search capabilities and prevents premature convergence, significantly enhancing optimization precision and convergence speed. Secondly, applying the CSRD-WOA algorithm to drone 3D path planning issues, the simulation results show that the CSRD-WOA algorithm can effectively manage path planning in complex terrains, showcasing its application potential in drone path planning.

## 1. Introduction

The rapid development of drone technology has brought revolutionary changes to various application fields, especially in precision agriculture [1], urban planning, and emergency rescue, where effective path planning is a key factor in ensuring efficient and safe task execution by drones [2]. Path planning directly affects task execution efficiency and drone energy management [3].

In recent years, numerous scholars both domestically and internationally have explored drone path planning methods and developed various algorithms. These algorithms are generally divided into two categories: traditional algorithms, such as the artificial potential field method [4] and the A * algorithm [4], and intelligent algorithms, such as the Particle Swarm Optimization algorithm [5], Grey Wolf Optimization algorithm [6], and the Whale Optimization Algorithm. The Whale Optimization Algorithm (WOA), proposed by Mirjalili and others [7], has proven superior to other algorithms in function optimization due to its simple principles, minimal parameter settings, and robust optimization performance, finding successful application in numerous fields, including engineering optimization and function maximization. However, the basic WOA has limitations in parameter control adjustment and maintaining population diversity, which restricts its balance between global search and local precision search, often leading it to fall prematurely into local optima [8]. Recent improvements to the Whale Algorithm include adding local search mechanisms and improving its convergence to adapt to more complex optimization problems [9]. These improvements demonstrate the feasibility and necessity of further optimizing the Whale Algorithm for applications such as path planning. Kun Wu and others [10] have used greedy optimization strategies to improve the Whale Algorithm, expanding its search range to enhance global optimization effects. However, this method is still limited by its convergence speed and optimization precision. Mehmet Enes Avcu [11] and others have applied the Whale Algorithm to drone path planning to avoid collisions in cluttered areas. Although effective, the optimization performance is still suboptimal and requires further improvement.

This paper proposes an improved Whale Algorithm that integrates Cuckoo Search [12] and Random Differential [13] strategies, ensuring the global search capability of the Whale Optimization Algorithm and its optimization precision. And the improved Whale Optimization Algorithm has been applied to drone 3D path planning for the first time.

## 2. Introduction to the Whale Optimization Algorithm

The Whale Optimization Algorithm (WOA) simulates the foraging behavior of whales, primarily involving two strategies: bubble-net feeding and random search.

### 2.1. Bubble-net encircling mechanism

The foraging behavior of humpback whales involves encircling prey and moving in a spiral to achieve local optimization. In the Whale Optimization Algorithm (WOA), each whale position in the search space represents a potential solution, where the individual closest to the optimal value of the objective function is considered the best. Other whales will update their positions by moving closer to this global best position, simulating the real-life behavior of whales encircling their prey can be described as shown in Equation (1):


$$\begin{array}{c} X\left(t+1\right)=X^{*}\left(t\right)-A\cdot D \end{array}$$ (1)

In the formula: $D=\left|C\cdot X^{*}\left(t\right)-X\left(t\right)\right|$; t represents the current iteration count. $X^{*}$ represents the global best position vector. *X* represents the current whale position vector.

A and C are parameter matrices, $A=2a\cdot r_{1}-a$, $C=2\cdot r_{2}$, $r_{1}$ and $r_{2}$ are random numbers between 0 and 1. $a=2-2t/t_{max}$, $t_{max}$ represents the maximum number of iterations. During the spiral update of positions, the distance between the current position and the best whale individual is first calculated, and then movement towards the best individual is performed in a spiral motion. The mathematical model for this phase can be expressed as shown in Equation (2):


$$X(\begin{array}{c} t+1) \end{array}=X^{*}\left(t\right)\;+D_{p}^{'}\cdot e^{bl}\cdot \cos\;\left(\begin{array}{c} 2\pi l \end{array}\right).$$ (2)

In the equation: $D_{p}^{'}=\left|X^{*}\left(t\right)-X\left(t\right)\right|$, *l* is a random number between -1 and 1, *b* is a constant which determines the shape of the helix.

When whales are within their enclosure, they approach their prey by feeding with bubble nets. In this case, when the amplitude of the spiral update is less than 1, the whale will perform a local optimal search while surrounding prey and performing spiral updates with a 50% probability. The mathematical model for this process is shown in Equation (3):


$$X\left(t+1\right)= \{\begin{array}{c} X^{*}\left(t\right)-A\cdot D\circ p<0.5\\ X^{*}\left(t\right)+D_{p}^{'}\cdot e^{bl}\cdot \cos(\begin{array}{c} 2\pi l \end{array})⩾0.5 \end{array}$$ (3)

### 2.2. Search and prey mechanism

In addition to the bubble-net method, whales also employ a random search strategy to locate prey. When the coefficient A exceeds the range of $\left[-1,1\right]$, the position of the whale is randomly updated based on the distance data D. This deviation from the original target enhances the algorithm’s global search capability, aiding in the search for solutions across a broader search space. As shown in Equations (4) and (5):


$$D=|C\cdot X_{\text{rand}}-X\left(j\right)|$$ (4)

$$X\left(j+1\right)=X_{\text{rand}}-A\cdot D$$ (5)

$X_{\text{rand}}$ Represents the position of a randomly selected individual whale from the current population.

## 3. Introduction to improvement strategy

### 3.1. Introduction to the random differential algorithm

The differential algorithm is an evolutionary optimization technique that mimics biological evolutionary mechanisms, specifically designed for optimizing continuous variables [14]. It continuously generates new candidate solutions through mutation and crossover operations, and evaluates the fitness values of these new solutions, retaining the optimal solution until the best result is achieved. In the random differential strategy, random elements are incorporated into the original differential algorithm. The specific formula is shown as Equation (6).


$$Y\left(t+1\right)=m_{1}\left(Y_{best}\left(t\right)-Y\left(t\right)\right)-m_{2}\left(Y^{*}\left(t\right)-Y\left(t\right)\right)$$ (6)

In the formula, $m_{1}$ and $m_{2}$ are random number between 0 and 1, $Y^{*}\left(t\right)$ represents a randomly selected individual.

By applying the random differential strategy to update positions again after an individual has updated its location, and retaining the best-performing position, this method can significantly prevent the population from being confined to local optima. Thus, the global search capability and optimization performance of the algorithm are enhanced.

### 3.2. Introduction to the Cuckoo Search

The Cuckoo Search strategy is inspired by the breeding behavior of cuckoos, utilizing strategies generated through Levy flights and random walks [15]. The execution of Levy flights is modeled by the following formula(7):


$$X\left(t+1\right)=X\left(t\right)+\alpha ⊕\text{Levy}\left(\eta \right).$$ (7)

In the formula, $X\left(t\right)$ is the t-th generation of the solution, $X\left(t+1\right)$is the $\left(t+1\right)th$ generation of the new solution. *α* is the step size coefficient, $\text{Levy}\left(\eta \right)$ is a random number drawn from the Levy distribution. The formula for $\text{Levy}\left(\eta \right)$ is shown in Equation (8).


$$\text{Levy}\left(\eta \right)∼\frac{\phi \times u}{|v{|}^{\frac{1}{\eta }}}$$ (8)

In the formula, *μ* and *ν* are random numbers uniformly distributed according to the normal distribution. *η* is the distribution parameter for Levy flights, typically set at 1.5.$\phi ={\left[\left(\text{Γ}\left(1+\eta \right)\sin\left(\frac{\pi \eta }{2}\right)\right)/\left(\text{Γ}\left(\frac{1+\eta }{2}\right)\eta \times 2^{\frac{\eta -1}{2}}\right)\right]}^{1/\eta }$, Γ is the Gamma function.

Since the algorithm generates new solutions around the existing best solution, Formula (9) can be rewritten as:


$$X\left(t+1\right)=X\left(t\right)+\alpha_{0}\times \left[\frac{\phi \times u}{|v{|}^{\frac{1}{\eta }}}\right]\times \left(X\left(t\right)-X_{best}\right)$$ (9)

$\alpha_{0}$is a scaling factor set to 0.01 in Formula (10), and $X_{best}$is the current optimal solution. Random walk is shown in Equation (10):


$$X\left(t+1\right)= \{\begin{array}{cc} \begin{array}{c} X\left(t\right)+r\left(X\left(m\right)-X\left(n\right)\right)\\ X\left(t\right) \end{array} & \begin{array}{c} r>P_{a}\\ otherwise \end{array} \end{array}$$ (10)

*r* is the probability of performing a Levy flight, and $P_{a}$ is a constant between 0 and 1. *m* and *n* correspond to different individuals.

### 3.3. Integration of Cuckoo Search and random differential strategy

Although the Cuckoo Search algorithm effectively enhances global search and prevents premature convergence into local optima through Levy flights, it is less efficient in adjusting local solutions [16]. In contrast, the Random Differential Algorithm maintains population diversity and effectively finds local optima through differential, crossover, and selection operations [17]. Therefore, combining these two algorithms can enhance global search capabilities, increase search randomness, and improve the quality of solutions and the convergence speed of the algorithm. This paper modifies Formula (10) to Formula (11) and names the improved Whale Algorithm as: Cuckoo Search-Random Differential Strategy [18] based Whale Optimization Algorithm (CSRD-WOA).


$$X\left(t+1\right)=\{\begin{array}{cc} X\left(t\right)+\alpha_{0}\times \frac{\phi \times u}{|v{|}^{\frac{1}{\eta }}}\times \left(X\left(t\right)-X_{best}\right) & r>P_{a}\\ r_{1}\left(X_{best}\left(t\right)-X\left(t\right)\right)-r_{2}\left(X^{*}\left(t\right)-X\left(t\right)\right) & otherwise \end{array}$$ (11)

### 3.4. Whale Optimization Algorithm with multi strategy fusion

In the CSRD-WOA, parameter selection significantly impacts algorithm performance. Key parameters have been designed as shown in Table 1, optimized based on extensive testing and sensitivity analysis to improve search efficiency and solution quality.

**Table 1:** Parameter settings.

Parameter	NP	dim	*t*_max_	b	*η*	*α*	Pa
Size	500	30	30	1	1.5	0.01	0.8

## 4. Algorithm performance testing and analysis

### 4.1. Test function setup

To fully validate the performance of CSRD-WOA, three types of test functions were selected: unimodal functions, multimodal functions, and fixed multimodal functions. Detailed descriptions are provided in Tables 2–4.

**Table 2:** Unimodal test functions.

Number	Expression	Function optimal solution
$F_{1}$	$f_{1}(x)=\sum_{i=1}^{n}{\left(x_{i}-z_{i}\right)}^{2}$	0
$F_{2}$	$f_{2}\left(x\right)=max\left\{|x_{i}|,1\leq i\leq 30\right\}$	0
$F_{3}$	$f_{3}(x)=\sum_{i=1}^{30}ix_{i}^{4}+random[0,1)$	0

**Table 3:** Multimodal test functions.

Number	Expression	Function optimal solution
$F_{1}$	$f_{4}(x)=\sum_{i=1}^{n}[x_{i}^{2}-10\cos(2\pi x_{i})+10]$	0
$F_{1}$	$f_{5}=-20\exp\left(-0.2\sqrt{\frac{1}{30}\overset{30}{\underset{i=1}{\sum }}x_{i}^{2}}\right)-\exp\left(\frac{1}{30}\overset{30}{\underset{i=1}{\sum }}\cos2\pi x_{i}\right)+20+c$	0
$F_{1}$	$\begin{array}{l} f_{6}(x)=\frac{1}{4000}\sum_{i=1}^{30}x_{i}^{2}{-\prod }_{i=1}^{30}\cos\left(\frac{x_{i}}{\sqrt{i}}\right)+1 \end{array}$	0

**Table 4:** Fixed multimodal test functions.

Number	Expression	Function optimal solution
$F_{1}$	$f_{7}(x)={\left(\frac{1}{500}+\sum_{j=1}^{25}\frac{1}{j+\sum_{i=1}^{2}{\left(x_{i}-a_{ij}\right)}^{6}}\right)}^{-1}$	0.998003837794450
$F_{1}$	$f_{8}(x)={\left(x_{2}-\frac{5.1}{4\pi^{2}}x_{1}^{2}+\frac{5}{\pi }x_{1}-6\right)}^{2}+10(1-\frac{1}{8\pi })\cos x_{1}+10$	0.397887357729738
$F_{1}$	$\begin{array}{l} f_{9}(x)=[1+{\left(x_{1}+x_{2}+1\right)}^{2}(19-14x_{1}+3x_{1}^{2}-14x_{2}+6x_{1}x_{2}+3x_{2}^{2})]\times \\ \left[30+{\left(2x_{1}-3x_{2}\right)}^{2}(18-32x_{1}+12x_{1}^{2}+48x_{2}-36x_{1}x_{2}+27x_{2}^{2})\right] \end{array}$	2.9999999999992

### 4.2. Comparative experiment

The CSRD-WOA algorithm is applied to the benchmark test functions described in Section 4.1, resulting in the statistical outcomes presented in Table 5.

**Table 5:** Benchmark test function result.

Function	Evaluation criteria	PSO	CS	WOA	CSRDWOA
F1	Best	372.3384	21.7327	0	0
	Mean	1906.5221	1221.8361	821.3629	212.7033
	Std	2490.9898	1786.2417	3501.8532	1549.3213
F2	Best	6.1713	12.4933	0.0693	0
	Mean	14.2241	22.76	12.9049	0.0009
	Std	6.456	9.6169	21.0429	0.0093
F3	Best	0.3997	0.0602	0.0033	0.0006
	Mean	2.8156	0.1771	0.5106	0.0045
	Std	9.9699	0.2356	2.5482	0.0284
F4	Best	221.4351	195.8508	119.215	3.4013
	Mean	28.9113	36.5113	57.1616	22.8166
	Std	14.5254	34.0211	61.1915	92.8571
F5	Best	5.5849	19.9387	5.06E-14	8.88E-16
	Mean	8.8591	19.9392	1.3684	0.0518
	Std	2.3626	0.0014	3.1324	0.4341
F6	Best	2.4873	1.0751	0.0222	0
	Mean	24.6904	8.8593	6.2936	0.3364
	Std	27.332	12.0609	28.8159	6.6783
F7	Best	2.9821	0.998	0.998	0.998
	Mean	3.0101	0.998	1.0718	0.998
	Std	0.439	0	0.2605	0
F8	Best	0.3979	0.3979	0.3979	0.3979
	Mean	0.3993	0.3979	0.3981	0.3979
	Std	0.0163	0	0.0014	0
F9	Best	3	3	3	3
	Mean	3.0495	3	3.002	3.0001
	Std	0.6172	0	0.0212	0.0016

**Fig 1:**
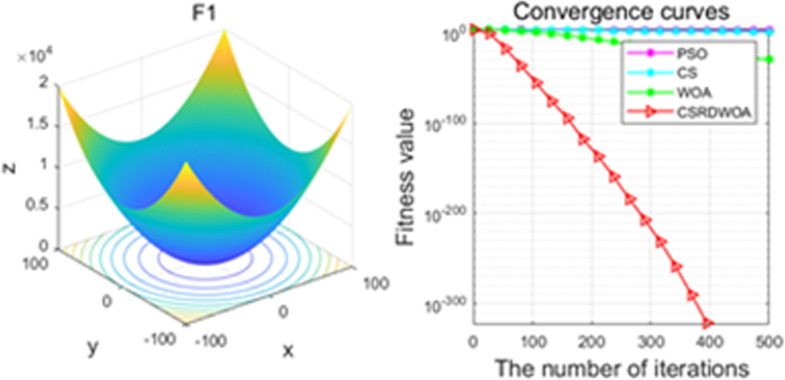
$F_{1}$.

**Fig 2:**
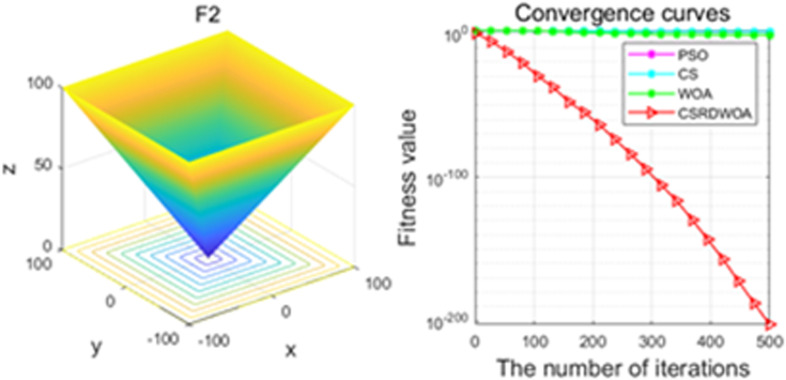
$F_{2}$.

**Fig 3:**
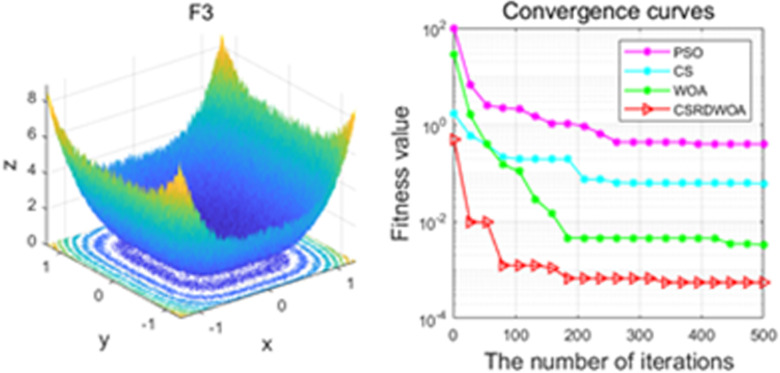
$F_{3}$.

**Fig 4:**
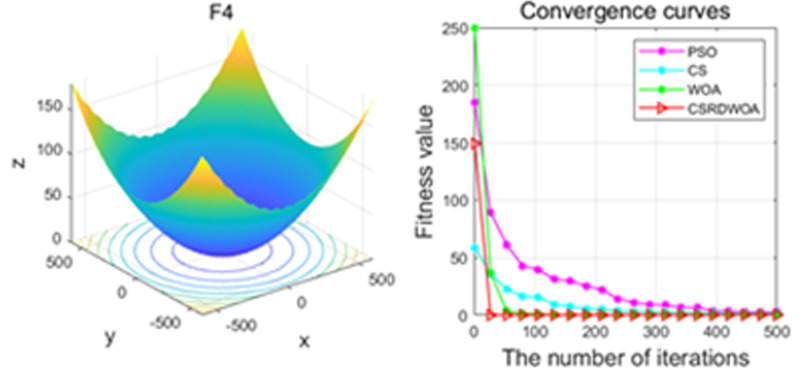
$F_{4}$.

**Fig 5:**
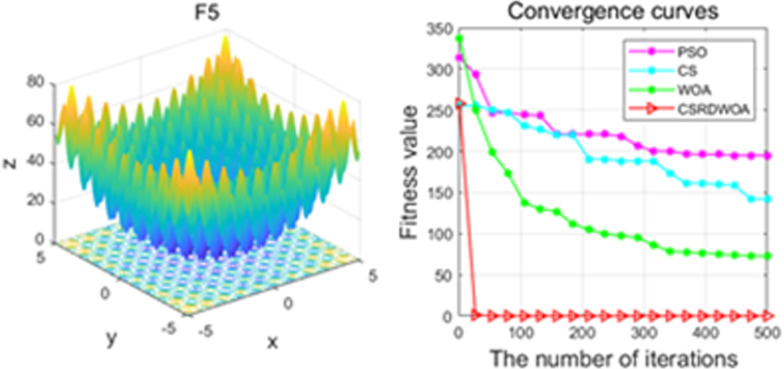
$F_{5}$.

**Fig 6:**
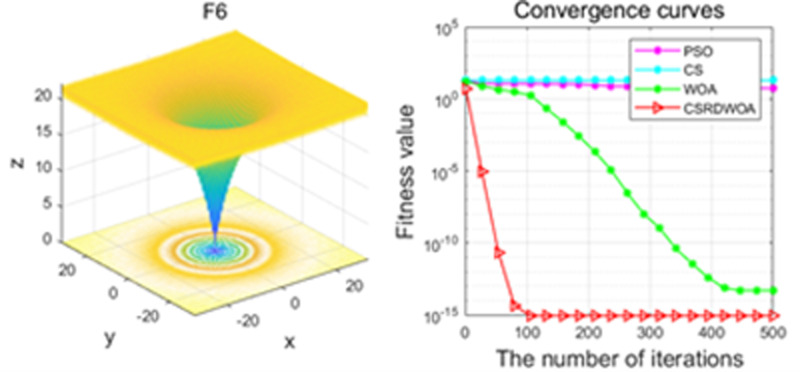
$F_{6}$.

**Fig 7:**
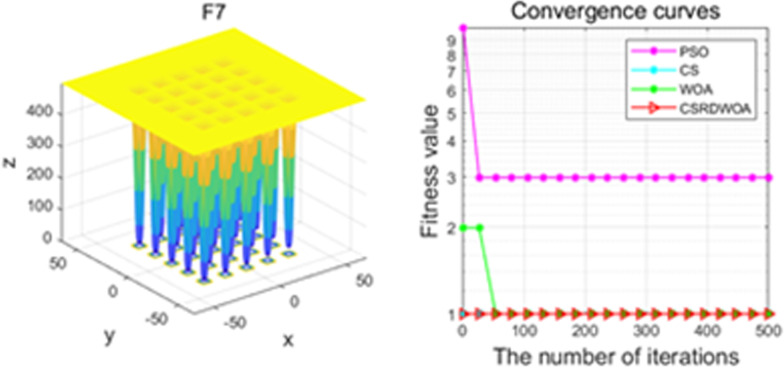
$F_{7}$.

**Fig 8:**
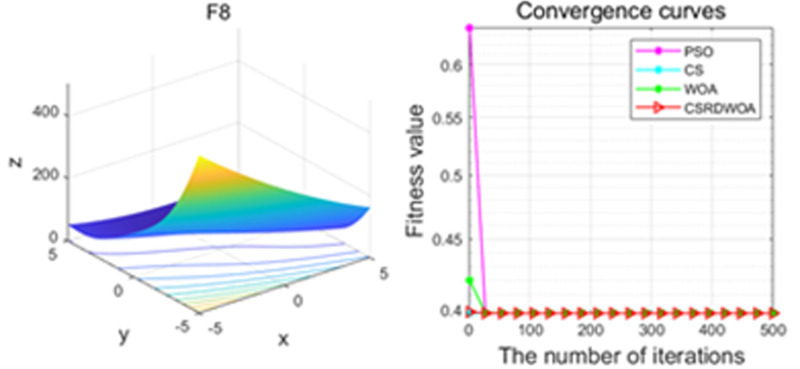
$F_{8}$.

**Fig 9:**
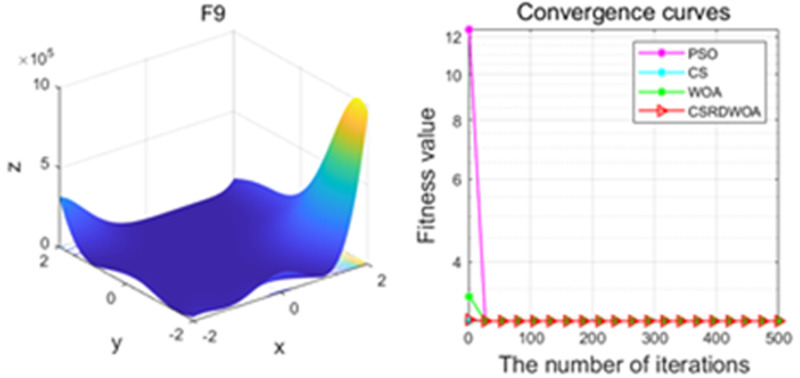
$F_{9}$.

Additionally, Figs 1–9 are displays images of several benchmark test functions. Among them, F1 and F2 are unimodal test functions, F4 and F5 are multimodal test functions, and F7 and F8 are fixed multimodal test functions. These three categories of test functions are used to evaluate function convergence, optimization capability, and the ability to handle complex problems, respectively. Based on the content of Figs 1–9 and Table 5, it is evident that the improved algorithm surpasses the original WOA in terms of convergence, optimization capability, and handling complex problems, indicating that the improvement strategies employed in this study are effective. Although the improved algorithm slightly underperforms the original WOA and PSO algorithms in some test results, the effectiveness of the adopted improvement strategies remains evident.

### 4.3. Ablation experiment

To demonstrate that the improvement strategies have enhanced the original WOA, this section employs ablation experiments [19] to verify the effectiveness of these strategies. The larger the obtained value, the higher the degree of improvement. The calculation formulas for the ablation experiment are shown in Equation (12).


$$\gamma_{1}=\frac{f_{CS—WOA}}{f_{WOA}}$$ (12)

$$\gamma_{2}=\frac{{f_{CS-WOA}}^{best}}{{f_{WOA}}^{best}}$$ (13)

The larger the obtained value, the better the improvement of the algorithm. In Formula (13),$f_{CS-WOA}^{best}$ and $f_{WOA}^{best}$ respectively represent the average best fitness values of the CSRD-WOA algorithm and the WOA algorithm. $f_{CS-WOA}^{best}$ and $f_{WOA}^{best}$ are the best fitness values of the CSRD-WOA and WOA algorithms on the test functions, respectively. Smaller values of $\gamma_{2}$ indicate poorer optimization performance of the control algorithm, highlighting that the missing strategy is crucial for enhancing the performance of the original algorithm. The results of the ablation experiment are shown in Table 6.

**Table 6:** Ablation experiment results.

Test function	*γ*_2_
F1	0
F2	7.9445e-196
F3	0.1666
F4	0
F5	0.0175
F6	0
F7	1
F8	1
F9	1

The results of the ablation experiments reveal that the improvement strategies adopted for CSRD-WOA enhance its performance compared to the original WOA. Although the improvements are not significant for some functions, the strategies can still be considered effective. Therefore, based on the results of the benchmark test functions and the ablation experiments, it can be concluded that the improvement strategies employed in this study have, to a certain extent, improved the performance of WOA.

## 5. Drone 3D path planning based on the improved Whale Optimization Algorithm

To verify the applicability of CSRD-WOA, the algorithm is applied to three-dimensional path planning for drones in complex environments [20]. The drone’s path planning needs to consider path cost, threat cost, flight altitude, and smoothness cost [21]. The total cost of drone flight is given by Equation (14).


$$F\left(X_{i}\right)=\sum_{k=1}^{4}b_{n}F_{n}\left(X_{i}\right)$$ (14)

Among them, X_i_ are the decision variables, b_n_ are the function weight parameters, and $F_{n}$ is the nth cost. The cost function is composed of three parts: the threat cost, the altitude cost, and the smoothness cost. Below, we will introduce these three cost functions.

### 5.1. Cost function

#### Threat environment cost.

**Fig 10:**
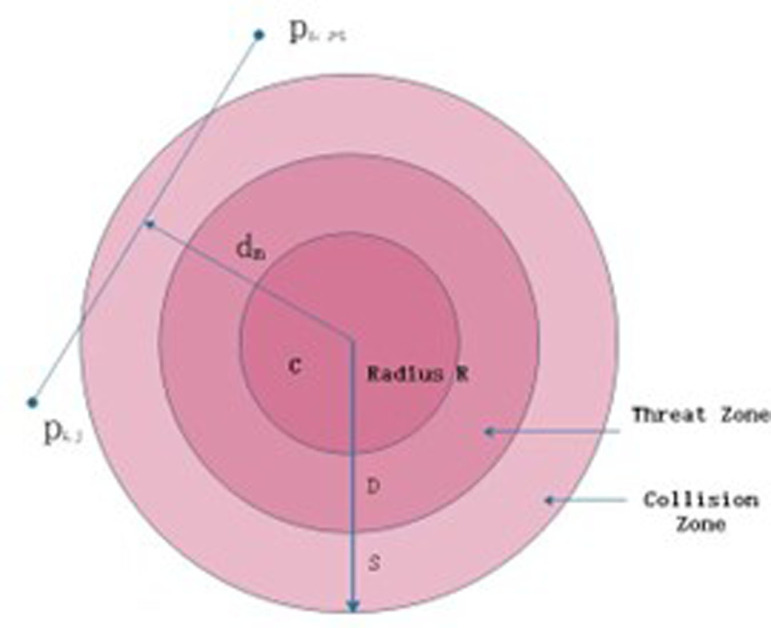
Threat area diagram.

Drone path planning takes safety into account, simplifying potential threats as fixed-radius cylindrical areas to optimize the route and ensure flight safety. As shown in Fig 10.

Let *M* be the set of cylindrical collections representing all potential threat obstacles, with the center of the base circle denoted by *C* and radius R. The diameter of the drone is *D*, and the vertical distance between adjacent path nodes is dim. *S* represents the range of the obstacle risk area. In order to quantify the safety of the drone’s path, the threat cost is calculated to evaluate the path risk, using Equation (15) to compute the threat cost:


$$\{\begin{array}{c} F_{1}\left(J_{i}\right)=\sum_{j=1}^{n-1}\sum_{m=1}^{M}T_{m}\left(P_{ij}P_{ij+1}\right)\\ T_{m}\left(P_{i,j}P_{i,j+1}\right)= \{\begin{array}{c} 0,d_{m}>S+D+R_{m}\\ \left(S+D+R_{m}\right)-d_{m},D+R_{m}<d_{m}\leq S+D+R_{m}\\ \infty ,d_{m}\leq D+R_{m} \end{array} \end{array}$$ (15)

When the drone is far from the danger zone, the threat level is zero; however, as it approaches the danger zone and the vertical distance (dm value) decreases, the threat correspondingly increases.

#### Flight altitude cost.

When a drone performs measurement and search tasks, its flight altitude is restricted by a minimum height $h_{min}$ and a maximum height $h_{max}$. A reasonable flight altitude ensures the safety of the drone’s flight and the precision of the collected images, optimizing task execution. The altitude cost is as shown in Equations (16) and (17).


$$H_{ij} \{\begin{array}{c} \left|h_{ij}-\frac{\left(h_{max}+h_{min}\right)}{2}\right|,h_{min}\leq h_{ij}\leq h_{max}\\ \infty ,otherwise \end{array}$$ (16)

$$F_{2}\left(X_{i}\right)=\sum_{j=-1}^{n}H_{ij}$$ (17)

The altitude cost aims to ensure that the drone remains within the set flight altitude, penalizing heights that exceed this range in order to reduce risks and improve efficiency.

#### Smoothness cost.

The drone’s heading is controlled by horizontal turning angles and vertical pitch angles, which need to be set appropriately to comply with flight restrictions and ensure path safety and efficiency in response to sudden and environmental challenges. The horizontal turning angle is the angle between continuous path segments on the horizontal plane. This angle is calculated using Equation (18):


$$\phi_{ij}=\arctan\left(\frac{∥\vec{P_{ij}^{'}P_{i,j+1}^{'}}\times \vec{P_{i,j+1}^{'}P_{i,j+2}^{'}}∥}{\vec{P_{ij}^{'}P_{i,j+1}^{'}},\vec{P_{i,j+1}^{'}P_{i,j+2}^{'}}}\right).$$ (18)

The vertical pitch angle refers to the angle difference between two consecutive path segments in the vertical direction, as shown in Equation (19):


$$\psi_{ij}=\arctan\left(\frac{z_{i,j+1}-z_{ij}}{∥\vec{P_{ij}^{'}P_{i,j+1}^{'}}∥}\right)$$ (19)

The calculation of the smoothness cost is as shown in Equation (20):


$$F_{3}\left(X_{i}\right)=a_{1}\sum_{j=1}^{n-2}\phi_{ij}+a_{2}\sum_{j=1}^{n-1}\left|\psi_{ij}-\psi_{i,j-1}\right|$$ (20)

In the equation, $a_{1}$ and $a_{2}$ are the penalty coefficients for the horizontal turning angle and the vertical pitch angle, respectively.

#### Flight distance cost.

The calculation of the flight distance cost involves measuring the displacement between adjacent data points along the drone’s flight path. By calculating the magnitude of the coordinate differences between these points, the length of each segment can be obtained. Summing these lengths yields the total flight distance cost. Assuming the three-dimensional coordinate data of the drone’s flight trajectory is $\left\{\left(x_{1},y_{1},z_{1}\right),\left(x_{2},y_{2},z_{2}\right),\ldots ,\left(x_{N},y_{N},z_{N}\right)\right\}$, whereN is the number of data points. The calculation formula for the flight path cost is shown in Equation (21).


$$F_{4}=\overset{N-1}{\underset{i=1}{\sum }}∥diff_{i}∥.$$ (21)

Based on the aforementioned four types of flight costs, the total cost of the drone flight can be determined by Equation (22).


$$F_{total}=\omega_{1}\times F_{1}+\omega_{2}\times F_{2}+\omega_{3}\times F_{3}+\omega_{4}\times F_{4}$$ (22)

In the equation, $\omega_{1},\omega_{2},\omega_{3},\omega_{4}$ respectively represent the weights for the threat environment cost, altitude cost, smoothness cost, and flight distance cost, with values set at: 10, 100, 10, 50. The flight cost parameters are as shown in Table 7. This weight coefficient value setting helps to provide a more intuitive observation of drone flight costs.

**Table 7:** Parameter settings.

Parameter	*S*	*D*	$a_{1}$	$a_{2}$	$\omega_{1}$	$\omega_{2}$	$\omega_{3}$	$\omega_{4}$
Size	20	10	1	1	10	100	10	50

### 5.2. Simulation testing and analysis

The simulation environment is configured on a Windows 10 system, using MATLAB 2019b to create two types of terrain: mountainous terrain with multiple structures and relatively flat mountainous terrain. Various threats are added to both types of terrain to simulate environments of different complexities for drone path planning experiments.

#### Case study one.

**Fig 11:**
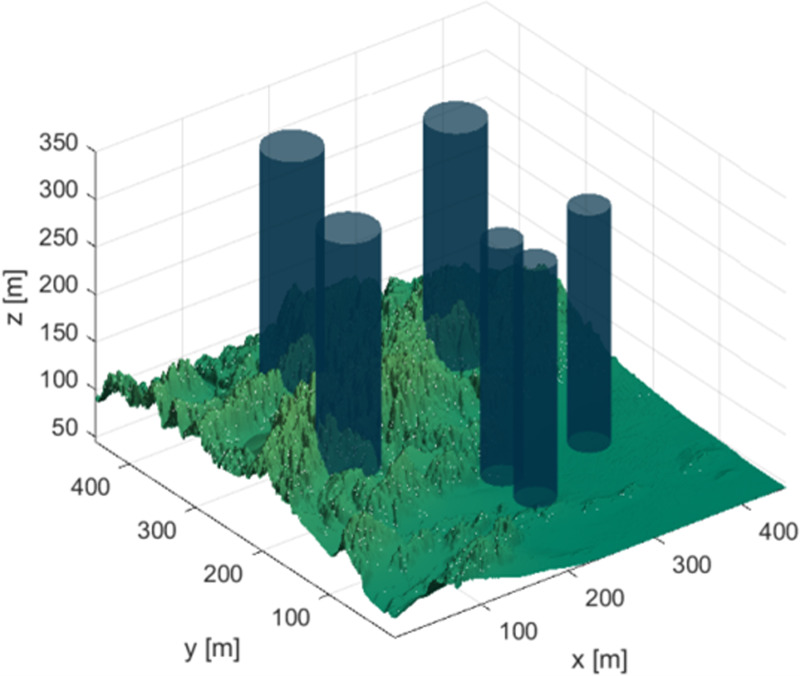
Terrain map for case study one.

***Terrain environment parameters*:** The terrain environment for Case Study One is shown in Fig 11. The deep blue cylinders represent the hazardous areas in the terrain. The test environment is set to an area of 400m by 400m, with a maximum flight altitude of 350m.

In the terrain scenario, the drone starts from the coordinates (10, 10, 200) m and flies to the endpoint at (400, 400, 150) m.

The coordinates of the hazardous areas in the terrain of Case Study One are shown in Table 8.

**Table 8:** Coordinates and radius of hazardous areas in case study one.

Serial number	Coordinates (unit: m)	Threat radius (unit: m)
1	(300, 300, 100) m	50 m
2	(200, 100, 100) m	40 m
3	(100, 200, 100) m	50 m
4	(300, 100, 100) m	40 m
5	(200, 50, 100) m	40 m
6	(150, 350, 100) m	50 m

**Fig 12:**
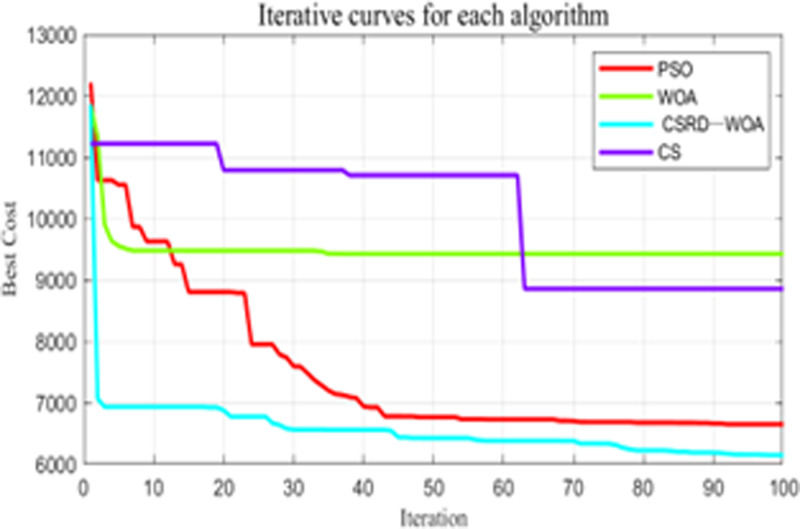
Flight optimization iterations.

***Simulation experiment*:** Comparative experiments are conducted using the CSRD-WOA algorithm proposed in this paper with the PSO algorithm, WOA algorithm, and CS algorithm. The resulting optimal fitness iteration curve for the flight is shown in Fig 12.

From the fitness curve diagram in Fig 12, it can be seen that the CSRD-WOA algorithm found the optimal solution. Additionally, the various costs associated with the droneflight are presented in Table 9.

**Table 9:** Flight cost.

Algorithm name	Optimal fitness value	Flight distance cost	Threat cost	Altitude cost	Smoothness cost
PSO	6648.9351	671.7923	5.3762e + 43	203.2753	278.7714
WOA	9422.5884	709.0645	6.9252	168.5617	0
CS	8853.7605	721.4416	5.3762e + 43	454.9298	329.5859
CSRD-WOA	6133.2575	600.1567	0	14.551	0

**Fig 13:**
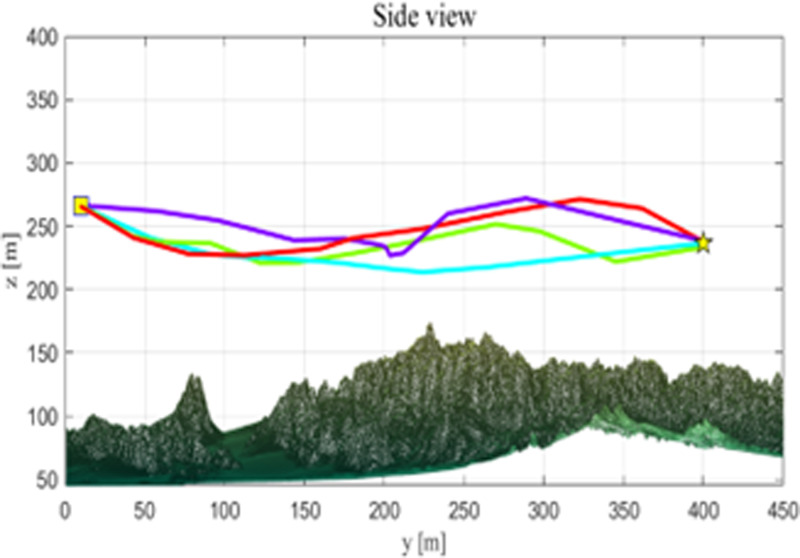
Side view.

**Fig 14:**
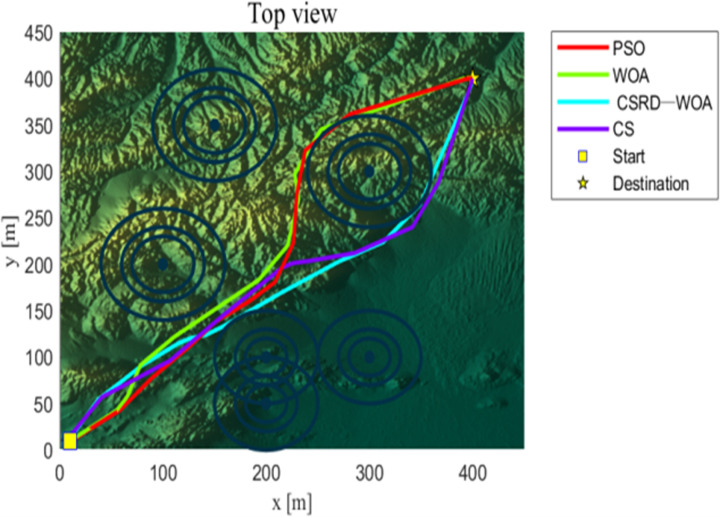
Top view.

**Fig 15:**
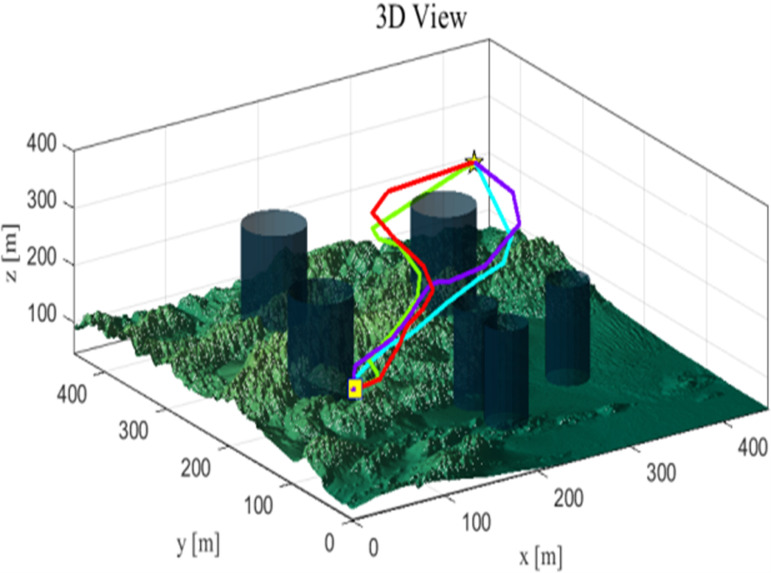
3D view.

As seen from Table 9, the CSRD-WOA algorithm achieved the optimal fitness value, and all associated costs are lower than those of the other algorithms. This indicates that the drone path planning optimized by CSRD-WOA can completely avoid threat areas while reducing flight costs. Figs 13–15 are shows the side view, top view, and 3D view of the flight path, respectively. These views illustrate the drone flight paths optimized by each algorithm, revealing that the path optimized by CSRD-WOA is safer and smoother.

#### Case study two.

To verify the accuracy and reliability of the experimental results, this study designed a series of new simulation experiments based on different ground conditions.

**Fig 16:**
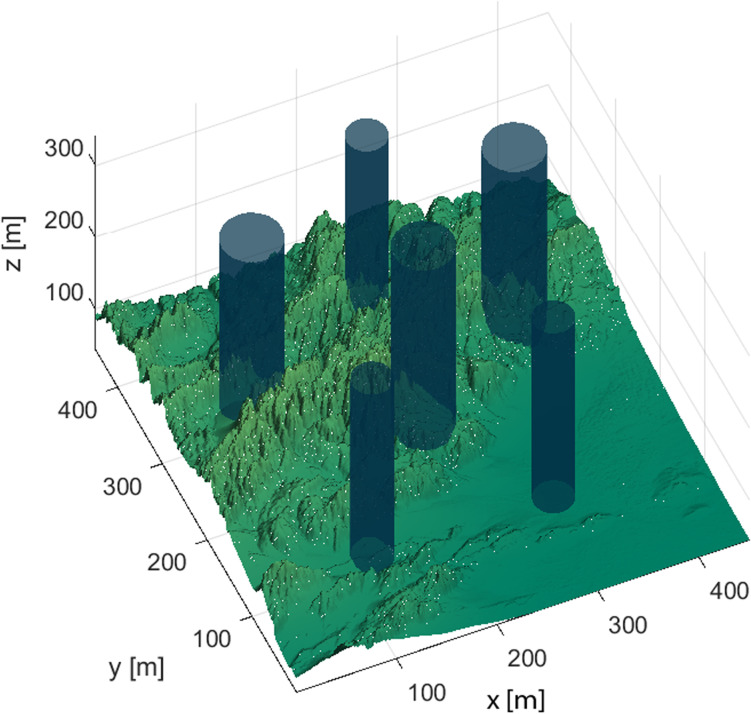
Terrain map for case study two.

***Terrain environment parameters*:** The terrain environment for Case Study Two is shown in Fig 16. The deep blue cylinders represent the hazardous areas in the terrain. The test environment is set to an area of 400m by 400m, with a maximum flight altitude of 350m. In the terrain scenario, the drone starts from the coordinates (10, 10,200) m and flies to the endpoint at (400, 400, 150) m.

The coordinates of the hazardous areas in the terrain of Case Study Two are shown in Table 10.

**Table 10:** Coordinates and radius of the hazardous area in case two.

Number,	Coordinates (unit: m)	Threat radius (unit: m)
1	(85, 290, 80)	30 m
2	(120, 100, 70)	20 m
3	(220, 210, 90)	30 m
4	(230, 360, 80)	20 m
5	(300, 100, 70)	20 m
6	(350, 300, 70)	30 m

**Fig 17:**
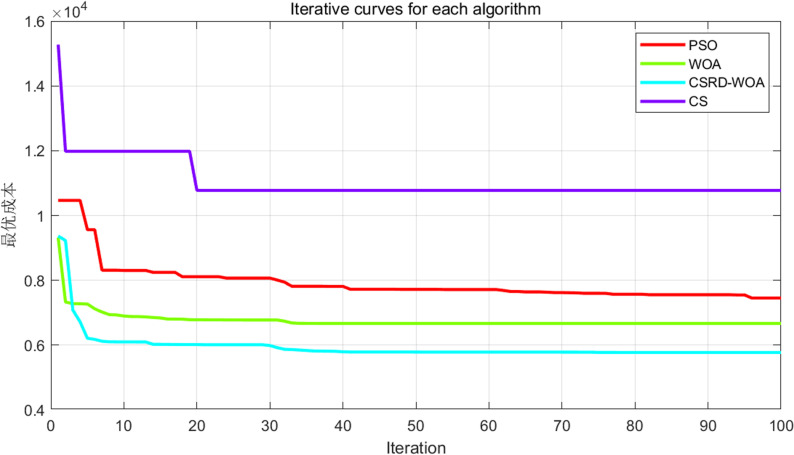
Flight optimization iterations.

***Simulation experiment*:** During the flight, the maximum turning angle of the drone is restricted to 45°. The simulation population size is 500, with a maximum of 100 iterations. The final optimal fitness for the flight, flight distance cost, threat cost, altitude cost, and smoothness cost are shown in Table 11, and the optimal fitness iteration curve for the flight is shown in Fig 17. Comparative experiments are conducted between the CSRD-WOA algorithm and the PSO algorithm, WOA algorithm, and CS algorithm.

**Table 11:** The cost of the flight in case two.

Algorithm name	Optimal fitness value	Flight distance cost	Threat cost	Altitude cost	Smoothness cost
PSO	7452.86	707.21	71.37	239.85	214.24
WOA	6669.11	604.91	0	62.00	0
CS	10775.75	681.65	2.69E + 43	217.30	514.07
CSRD-WOA	5773.15	577.31	0	0.0035	0

**Fig 18:**
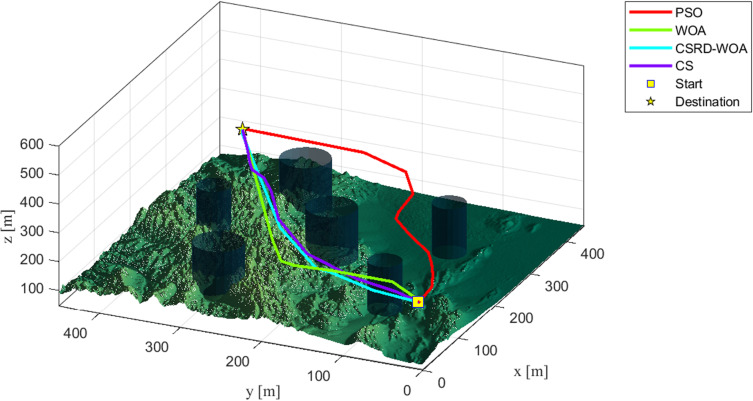
3D view.

**Fig 19:**
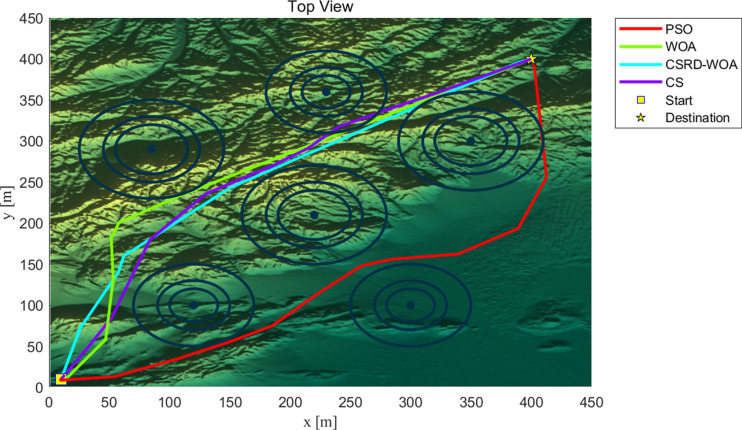
Top view.

**Fig 20:**
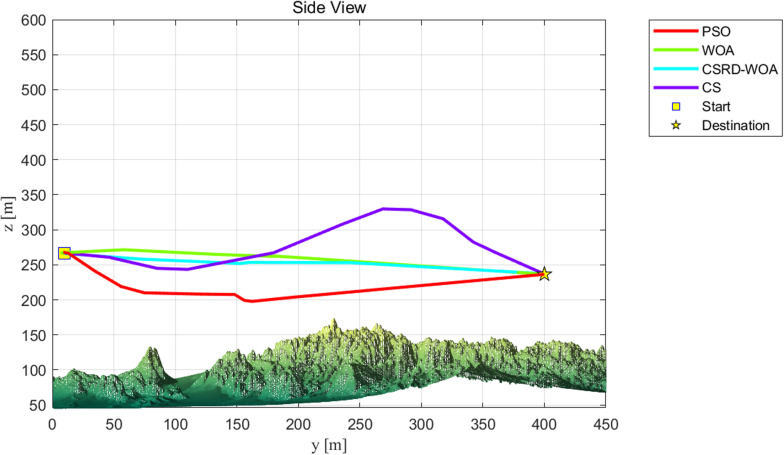
Side view.

From the fitness curve diagram in Fig 17, it can be seen that the CSRD-WOA algorithm found the optimal solution, while the CS algorithm, WOA algorithm, and PSO algorithm all fell into local optima. This indicates that the precision of the CS, WOA, and PSO algorithms in finding the optimal solution is far inferior to that of CSRD-WOA. As seen from Table 11, the CSRD-WOA algorithm outperforms the other three algorithms in total cost, flight distance cost, threat cost, altitude cost, and smoothness cost. This suggests that the CSRD-WOA algorithm is more suitable for finding the optimal path for drones. Furthermore, the flight path of CS passes through the threat area, which is very disadvantageous for the safety of drone flight. Figs 18–20 are shows the side view, top view, and 3D view of the flight path, respectively.

From the 3D visual diagram, top view, and side view of the flight paths shown in Figs 18–20, it can be observed that the drone’s trajectory based on the CSRD-WOA algorithm is straighter, shorter, smoother, and successfully avoids all hazardous areas.

In summary, in both scenarios, CSRD-WOA demonstrates higher efficiency in avoiding various threat terrains, and it achieves the lowest total cost, flight distance cost, threat cost, altitude cost, and smoothness cost in path planning.

## 6. Conclusion

This paper proposes a three-dimensional path planning method for drones based on the CSRD-WOA algorithm. Firstly, after the completion of iterations in the whale algorithm, the diversity and randomness of the population are increased using the strategy of cuckoo-random differential, which compensates for the drawback of the population being prone to local optima in the early stage. This strategy allows the population to escape local optima and improves global search capability. Secondly, a model of drone terrain, threat sources, and drone constraints is constructed, and corresponding cost functions are defined. Finally, simulation results show that the CSRD-WOA algorithm performs excellently in drone path planning, enabling the drone to quickly traverse dangerous areas and avoid obstacles within the shortest distance, demonstrating significant practical value.
